# Case of Phthisis Pulmonalis, Successfully Treated by Digitalis

**Published:** 1803-12-01

**Authors:** 

**Affiliations:** Featherstone Street, Finsbury


Case of Phthisis Pulmonalis, *successfully treated by Digitalis; communicated by* Mr. Dunn, *of Featherstone Street, Finsbury.*

ELIZABETH COULSTON, aged ten years and a half, of a delicate habit, about five or six weeks from the commencement of this history, had been attacked with fever, and which, as far as I could learn, was of an inflammatory nature. The medical gentleman who attended, bled her two or three times and administered nitrous medicines; the disease however continued to increase, and an affection of the lungs took place, plainly evincing a translation of morbid matter to those organs. Blisters were applied and nitre given, but to little purpose. The mother, who was but a poor woman, and could ill afford the attendance of an apothecary, where there was scarce any prospect of relief, and finding the child getting worse instead of better, declined his farther assistance. In this state she remained for better than a week, when I was desired to see her, and the following is an accurate statement of the condition in which I found her.

June 24, 1803. For several days past she had taken no medicine except a nitrous powder or two, the reliques of those which the gentleman who had attended had given her. The symptoms were, violent cough, attended with great pain and a spasmodic action of the diaphragm, drawing in the scrobiculus cordis during the fit, accompanied with pain; a considerable rattlihg noise in the throat, as if the bronchiæ were choaked up with matter; expectoration of a great quantity of thin matter; great emaciation; yellow countenance; facies hippocratica; the body reduced to a mere skeleton; hectic flushes; colliquative diarrhœa; dyspnœa; pulse quick and hard; violent pulsation of the carotids, which could be distinctly seen at a considerable distance beating with force; loss of appetite, pain, and inability of lying on the left side; with these symptoms were conjoined extreme weakness and faintings, insomuch that she could not stand or sit up without being supported.

Attentively considering all the symptoms, and the state of the pulse, I determined within my own mind that there was not a moment to be lost, and that energetic methods should be immediately adopted. The digitalis occurred to me, and I thought this a favourable opportunity for trying its effects, at the same time that I had more hopes from it, than from any thing else I could think upon.

I began by giving tinct. digitalis gt. ij. every four hours, in an oleaginous mixture, with a small quantity of nitre in it.

June 25. Cough somewhat relieved; pain, expectoration, diarrhœa, and other symptoms much the same, the pulse rather slower if any thing; but no considerable alteration could be expected in so desperate a case in such a short time. Tinct. digitalis gtt. iij. every four hours in the same kind of mixture.

26th. Cough, diarrhœa, and expectoration, somewhat less. Pulse less frequent; hectic flushes continued, particularly in the morning and evening.

27th. Cough considerably abated, diarrhœa almost gone, expectoration less and thicker; hectic flushes the same, feet and ancles considerably swelled, pain across the chest gone; dyspnœa and rattling in the throat greatly relieved. Tinct. digitalis gtt. iv. 4tis horis sumendæ.

28th. Towards the afternoon yesterday the cough became worse and more frequent, attended with pain and drawing in of the umbilicus, which abated in the evening, on the coming on of the hectic paroxysm. This morning more cheerful, less fever and cough; had expectorated since yesterday morning a great deal of tough, viscid phlegm, consisting of fibres or threads; complained of an itching in the nose and about the anus, with costiveness. Suspecting there might be some worms in the intestines, I gave the following. ℞. Pulv. rhæi gr. viij. calomel gr. ij. aloes socot. gr. ij. M. ft. pulv. vesperi sumendus. Cont. tinct. digitalis gtt. iv. 4tis horis in the same mixture.

29th. The powder operated five or six times, and brought off a quantity of dark coloured fæces, but no worms could be perceived; which however might, if they existed, have been indiscoverable in consequence of the blackness of the stools. Cough very violent, expectoration increased, violent cramps in the legs during the night, spasmodic contraction of the extensor tendons of the feet and toes, feet swelled, periodical hectic flushes, accompanied with cough, recurring every four or five hours. Gave her a spermaceti mixture with a few drops of tinct. opii; and gave tinct. digitalis gtt. vj. 4tis horis.

30th. Much better; all the symptoms relieved; more cheerful; had slept a little; pulse about 70; cough and expectoration moderated very much; bowels pretty regular. ℞. Digitalis gt. ix. 4tis hortis.

July 1. Cough and expectoration diminished, and the paroxysms shortened; the drawing in of the umbilicus, which which had been all along productive of great pain and distress in coughing was also lessened. Speech more clear and distinct, hollow rattling in the throat quite gone, appetite somewhat returned; she drank milk and ate light puddings, toasted bread, vegetables, &c. Tinct. digitalis, gtt. xij. 4tis horis.

2d. Continues to get better; all the symptoms considerably abated, speech very distinct, cough very little, expectoration small in quantity and thick, pulse more slow and not so hard. Tinct. digitalis as yesterday.

3d. Much as yesterday, but complains of great faintness and inconvenience from the excessive heat of the weather. Tinct. digitalis gtt. xiv. 4tis horis.

4th. Continues very weak and faint, cough and expectoration very little; extreme debility the most troublesome symptom now remaining, the others being almost entirely removed.

5th. Pains in the abdomen about the umbilicus, at times very severe; the digitalis had occasioned some nausea and giddiness, diminished therefore to twelve drops to be given occasionally with the spermaceti mixture, when the cough was troublesome. As the chief complaint consisted in great weakness, I gave the bark with a small quantity of steel. ℞. Pulv. cinchon. ʒij. aq. menth. pip. ℥iv. tinct. ferr. muriat. gtt. xx. capt. cochl. 1. ter in die.

6th. Better; pains gone off; continued the same plan as yesterday.

7th. Cough and expectoration almost entirely vanished; something stronger; continued the bark and digitalis occasionally from this time to the

13th. Nothing occurred worthy of recording; there was a little return of cough now and then, when the balsamic mixture with the digitalis was had recourse to; the heat of the weather oppressed her very much; debility still considerable; appetite increased.

20th. Continued in a convalescent state, though from the debility and still continued excessive heat of weather, she regained her strength but slowly.

The pain and inability of lying on the left side (the lung, I imagine, to be the most affected) was very nearly gone, as well as the cough.

She continued to take the bark and stomachic bitters, with the use of the digitalis, on the recurrence of the cough, for a short time; she got gradually better, and is now a fine hearty plump girl, full of health and spirits.

This is a true narrative, copied from the notes which I

daily took of the progress of the disease during the time I was attending the patient; and I think every one must admit the rapid recovery of this girl was entirely owing to the effect of the digitalis. Though given in small doses, I adjusted it as nearly as possible to the state of the system, which was in a high state of irritability; and the increase even of two drops sometimes occasioned, as I have instanced, nausea and giddiness.

Should the Case tend to confirm the effect of digitalis in cases of phthisis, and increase its partizans, my intention in recording it is answered; as I think it certainly, if not immediately cure the disease, will in most cases so considerably diminish the vascular action, as to allow Nature, by her operations, to restore the diseased lungs to their former state, and to more healthy actions.

The tinctura digitalis which I made use of, was made as follows, according to Dr. Latham: R. Digitalis sicca et contusa, ℥j. spt. vini tenuis, ℔j. Digere per dies octo et cola.

*October* 12, 1803.

